# Nuclear Envelope Transmembrane Proteins in Myotonic Dystrophy Type 1

**DOI:** 10.3389/fphys.2018.01532

**Published:** 2018-10-30

**Authors:** Stefan Hintze, Lisa Knaier, Sarah Limmer, Benedikt Schoser, Peter Meinke

**Affiliations:** Friedrich-Baur-Institute at the Department of Neurology, University Hospital, Ludwig-Maximilians-University Munich, Munich, Germany

**Keywords:** myotonic dystrophy type 1, nuclear envelope, nuclear envelope transmembrane proteins, myoblasts, myotubes

## Abstract

Myotonic dystrophy type 1 (DM1) is a multisystemic disorder with predominant myotonia and muscular dystrophy which is caused by CTG-repeat expansions in the *DMPK* gene. These repeat expansions are transcribed and the resulting mRNA accumulates RNA-binding proteins involved in splicing, resulting in a general splicing defect. We observed nuclear envelope (NE) alterations in DM1 primary myoblasts. These included invaginations of the NE as well as an altered composition of the nuclear lamina. Specifically, we investigated NE transmembrane proteins (NETs) in DM1 primary myoblasts, staining to determine if their distribution was altered compared to controls and if this could contribute to these structural defects. We also tested the expression of these NETs in muscle and how localization changes in the DM1 primary myoblasts undergoing differentiation in vitro to myotubes. We found no changes in the localization of the tested NETs, but most tended to exhibit reduced expression with increasing *DMPK*-repeat length. Nonetheless, the DM1 patient expression range was within the expression range of the controls. Additionally, we found a down-regulation of the possible nesprin 1 giant isoform in DM1 primary myoblasts which could contribute to the increased NE invaginations. Thus, nesprin 1 may be an interesting target for further investigation in DM1 disease pathology.

## Introduction

Myotonic dystrophies (DM) are caused by repeat expansions in non-coding regions of the *DMPK* (type 1, DM1) and *CNBP* (type 2, DM2) genes. The transcribed, repeat-containing RNA forms hairpin structures which yield a strong interaction with certain RNA binding proteins. Patient cells accumulate foci containing this RNA and its interacting proteins. Amongst these interacting proteins are MBNL-proteins, which are involved in the regulation of alternative splicing. The accumulation of these proteins on the repeat RNA leads to them not being available for normal splicing so that a general mis-splicing (“spliceopathy”) appears to occur with a shift to embryonic splice variants.

Clinically, DM is a progressive multisystemic disorder characterized by myotonia, muscle weakness, cataracts, and cardiac arrhythmia that can evolve to cardiomyopathy, insulin insensitivity and diabetes, testicular failure, and hypogammaglobulinemia (Udd and Krahe, 2012; Wenninger et al., 2018). The predominant muscle involvement brands DM as the most frequent muscular dystrophy in adulthood. Expansions of a CTG repeat in the 3' UTR of the *DMPK* gene cause DM1 (Fu et al., 1992). Up to 35 of these CTG repeats are considered to be normal, 35 to 49 repeats are a premutation, and 50 or more CTG triplets are considered to be disease causing. There is a rough correlation between repeat tract length and disease severity in DM1. The longer the CTG repeats the more severe the disease. Between 50 and ~150 repeats have been observed in patients with mild phenotype and ~100 to ~1000 repeats were identified in patients with classical DM, while more than 1000 CTG-triplets result in congenital DM, the most severe form of the disease (De Antonio et al., 2016).

Muscle differentiation defects have been described for DM1 (Furling et al., 2001; Mastroyiannopoulos et al., 2008), but it is unclear if the observed nuclear envelope alterations in DM1 myoblasts (Meinke et al., 2018) contribute to these defects in a similar manner as they do to nuclear envelope linked disease (Meinke and Schirmer, 2016). The most visible NE alterations are invaginations, which have been previously observed in DM1 fibroblasts (Rodriguez et al., 2015), and down-regulation of the lamins A and B1 (Meinke et al., 2018). We found a correlation between *DMPK* repeat length and the number of nuclei with NE invaginations (Meinke et al., 2018). With the aim to investigate which factors—apart from lamins—contribute to these structures, we screened a set of selected NE transmembrane proteins (NETs) for altered distribution in DM1 patients.

NETs have been linked to a wide range of disorders which include several myopathies (Meinke and Schirmer, 2016). In the light of the NE aberrations observed in DM1 myoblasts and myotubes (Meinke et al., 2018), NETs are possible candidates to contribute to DM1 muscle pathology. We decided to test the proteins emerin, LBR, TMEM38a, TMEM70, SUN1, SUN2, nesprin 1, and nesprin 2 for their localization and expression in primary DM1 myoblasts compared to controls. All of these proteins are NETs expressed in muscle (Wilkie et al., 2011; Korfali et al., 2012). Of these selected NETs, muscular dystrophies are linked to emerin (Bione et al., 1994), nesprin 1 and nesprin 2 (Zhang et al., 2007), as well as SUN1 and SUN2 (Meinke et al., 2014). SUN1, SUN2, nesprin 1, and nesprin 2 are all core components of the LINC (linker of nucleo- and cytoskeleton) complex (Crisp et al., 2006), while emerin is also involved in connecting the nucleus to the cytoskeleton (Salpingidou et al., 2007). The NETs Tmem38a and LBR are involved in genome organization (Holmer and Worman, 2001; Robson et al., 2016). Nesprins1 and 2 encompass several proteins due to having many splicing isoforms, some of which are specifically up-regulated during muscle differentiation (Duong et al., 2014).

Further indication for a possible NE involvement comes from myotonic dystrophy protein kinase (DMPK), the protein encoded by the *DMPK* gene, which has been reported to localize to the NE. DMPK has been identified at the NE in HeLa cells as well as in C2C12 mouse myoblasts and neonatal rat cardiac myocytes (Harmon et al., 2008, 2011) and reduced DMPK levels in DM1 patients have been observed (Fu et al., 1993). However, if haploinsufficiency of DMPK is a relevant factor in DM1 pathology remains unclear as two different DMPK-knockout mouse models show either a late onset progressive myopathy or no muscular phenotype at all (Reddy et al., 1996; Carrell et al., 2016).

## Materials and methods

### Patients and controls

Primary human myoblasts were obtained from the Muscle Tissue Culture Collection (MTCC) at the Friedrich-Baur-Institute (Department of Neurology, Ludwig-Maximilians-University, Munich, Germany). All control and patient materials were obtained with written informed consent of the donor. Ethical approval for this study was obtained from the ethical review committee at the Ludwig-Maximilians-University, Munich, Germany (reference 45–14). Repeat length was diagnosed on DNA extracted from blood. DM1 patients had the following DMPK repeat lengths: DM1-1 240-430; DM1-2 400-600; DM1-3 1100-1300; DM1-4 1500. The patient cell lines used are identical to those where a repeat-length depending enrichment of nucleoplasmic reticuli was seen (Meinke et al., 2018).

### Tissue culture

Myoblasts were grown in tissue culture using skeletal muscle cell growth medium (PeloBiotech, Munich, Germany). Myoblasts were kept from reaching confluency to avoid differentiation. Passage numbers were matched for controls and patient cells for the respective experiments, throughout all experiments passage numbers 8 to 10 have been used. Coverslips for myoblast immunohistochemistry were fixed at about 60–70 % confluence. Cell lysates for Western blot were taken at the same confluence, all at an average of 3 days of culturing after the last splitting. All samples were taken at least two passages after thawing.

For differentiation confluent myoblasts were cultivated for 7 days in DMEM containing 5% horse serum.

### Immunohistochemistry

Myoblasts were fixed with methanol (−20°C). Primary antibodies used for staining include: lamin A/C 4A7, emerin 5D10, nesprin 1 8C3 (provided by Glenn E. Morris), nesprin 1, nesprin 2 (provided by Didier Hodzic), Sun1 Atlas (HPA008346), Sun2 11208, LBR 11745, TMEM70 (provided by Eric C. Schirmer) and TMEM38a Milipore (06-1005). All secondary antibodies were Alexafluor conjugated. DNA was visualized with DAPI (4,6-diamidino-2 phenylindole, dihydrochloride).

### Microscopy and image analysis

All images were obtained using an Olympus FluoView FV1000/BX 61microscope equipped with a 1.42 NA 60x objective and 3x zoom magnification. Image analysis was performed using ImageJ software.

### Protein extracts

Myoblasts were washed with 1x PBS and trypsinized. Cell suspensions were inactivated with 10% FCS containing DMEM, collected in 1.5 ml tubes, centrifuged and washed twice in PBS. Cell pellets were directly dissolved in 10 mM Tris/HCl pH 7.6 1 % SDS lysis buffer containing protease inhibitors (cOmplete Tablets EDTA-free Roche 04 693 132 001).

For myotube extracts we first separated the myotubes using a minimal trypsin treatment to detach myotubes that were then gently centrifuged to sediment myotubes and separate them from remaining mono-nucleated cells: after this we used the same protocol as for myoblasts.

### Western blotting

Proteins from whole protein extracts were separated by SDS gel electrophoresis using 4–15% TGX (BioRad #456-8087) and self-prepared gels. Western blotting was performed using the Trans-Blot® TurboTM system (BioRad). Proteins were transferred to low fluorescent PVDF membranes (part of Trans-Blot® TurboTM RTA Transfer Kit #170-4274). Membranes were blocked with 5% BSA or 5% skim milk in 1xTBS/0.1% Tween®20. Primary antibodies used were: lamin A/C 4A7, emerin 5D10, nesprin 1 8C3 (provided by Glenn E. Morris), nesprin 1, nesprin 2 (provided by Didier Hodzic), Sun1 Atlas (HPA008346), Sun2 11208, LBR 11745, TMEM70 (provided by Eric C. Schirmer), and TMEM38a Millipore (06-1005). As loading controls for quantification, mouse anti-GAPDH (Milipore #MAB374), rabbit anti-GAPDH (Cell Signaling D16H1; XP #5174) and goat anti-GAPDH (Thermo PA1-9046) were used. Secondary antibodies used were: donkey anti-mouse IRDye 680RD, donkey anti-mouse IRDye 800CW, donkey anti-rabbit IRDye 680RD, donkey anti-rabbit IRDye 800 CW, and donkey anti-goat HRP (Dianova 705-035-003). For chemiluminescence detection we used Weststar Supernova (Cyanagene XLS3). All Western blot images were obtained using a Licor FC. Quantification was done using the Licor ImageStudio Software. Western blots were repeated at least twice to confirm the results (Figure S1: complete blots).

## Results

**Figure 1:**
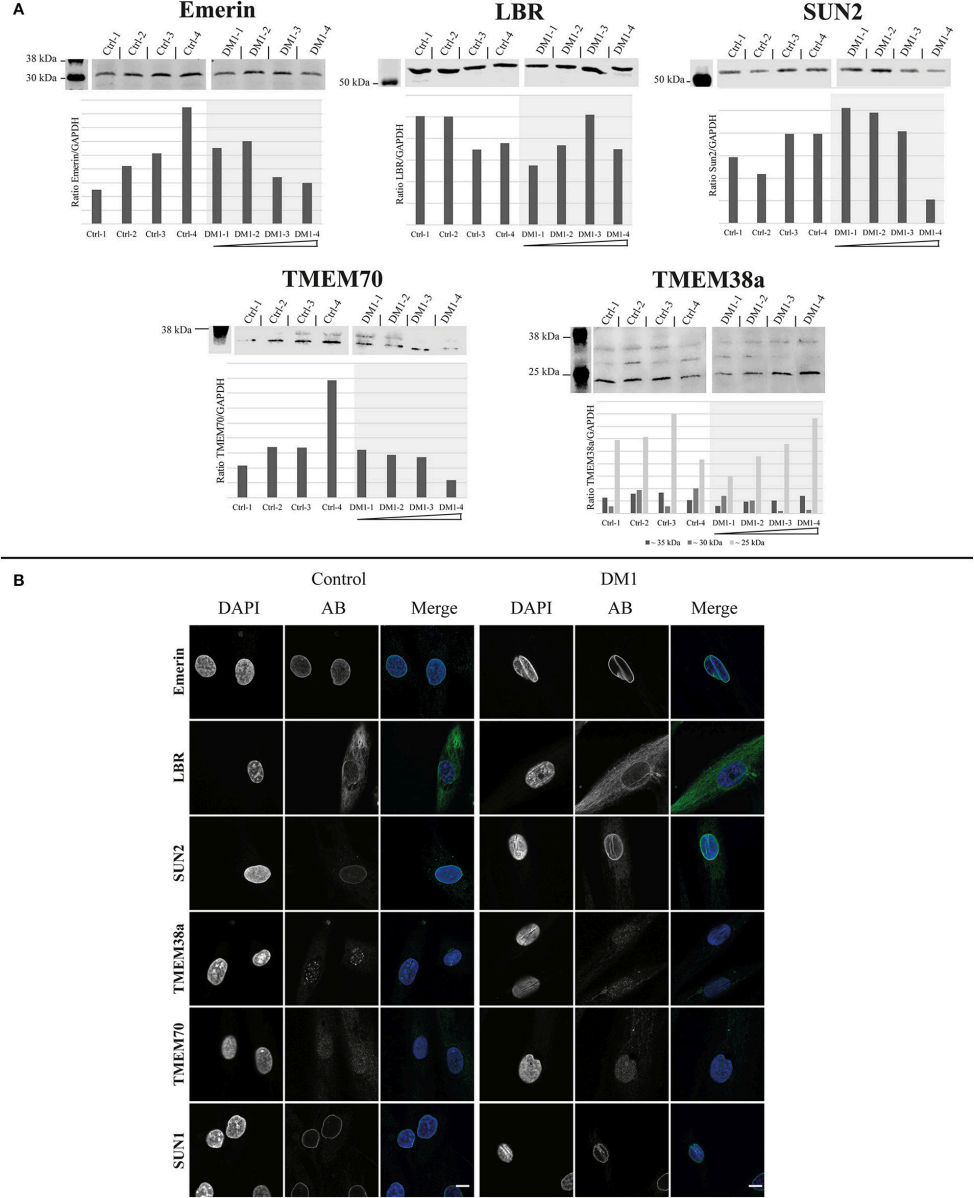
Expression and localization of nesprin1 and nesprin2 primary myoblasts and myotubes. Western Blot, quantification and immunofluorescence staining of primary control and DM1 myoblasts and myotubes for nesprin1 **(A)** and nesprin2 **(B)**. DM1 samples are ordered according their diagnosed repeat length from left (small repeat) to right (long repeat). Scale bar 10 μm. The lanes for DM1-3 and DM1-4 have been rearranged for the nesprin1 blots to allow a presentation according to repeat length (indicated by black boxes). For original blots see supplemental material.

First, we tested the expression levels of the NETs emerin, LBR, SUN2, TMEM70, and TMEM38a in four DM1 patient-derived primary myoblasts of different *DMPK* repeat length and compared them to four controls (Figure 1A).

The range of emerin expression levels within the set of patient-derived myoblasts is no greater than the expression range of the controls. This is also the case for LBR (Figure 1A). However, for SUN2 expression decreased with increasing repeat length. Nonetheless, compared to controls these changes seem to be within the normal expression range for SUN2. The same tendency can be observed for TMEM70; also here the expression is within the control range. For TMEM38a we see a tendency for the ~35 kDa band to increase with increasing repeat length, but also without deviation from the control range.

The immunofluorescence stainings for these NETs show, similar to the Western blots, no big differences between patients and controls in their localization (Figure 1B). Emerin, LBR, SUN2, and SUN1 antibodies stained the NE in both patients and controls. In addition, NE invaginations were stained by these four proteins in the patient samples. For TMEM38a and TMEM70 we did not observe NE staining in either patient or control myoblasts, consistent with their induction and NE localization occurring during myogenesis (data not shown). The only difference is that in the controls lines we see spots within the nucleus stained by the TMEM38a antibody. These spots seem to be reduced in patient cells (Figure 1B).

**Figure 2:**
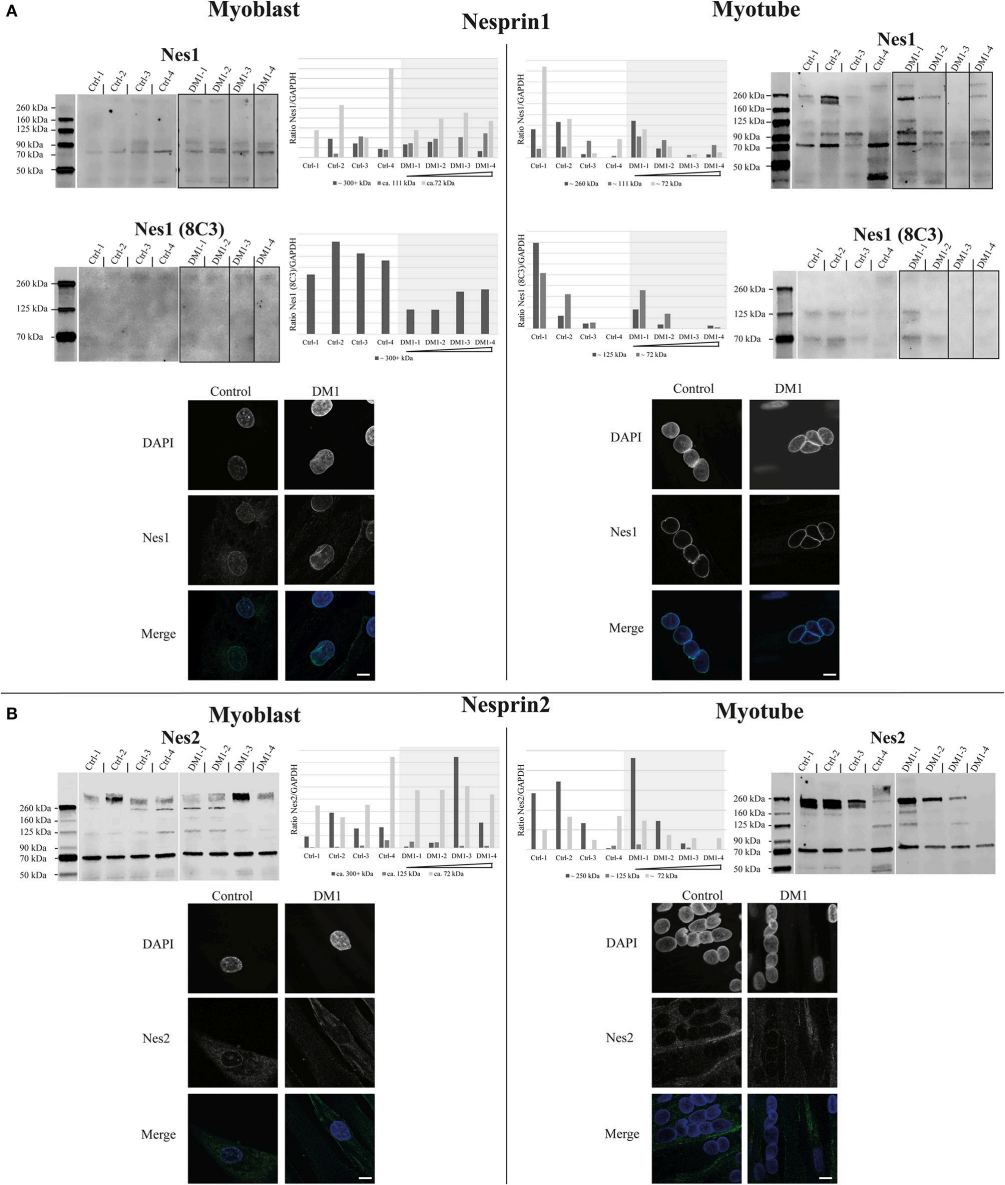
Expression and localization of different nuclear envelope proteins in myoblasts. **(A)** Western Blot and quantification of primary control and DM1 myoblasts for emerin, LBR, Sun2 TMEM70, and TMEM38a. DM1 samples are ordered according their diagnosed repeat length from left (small repeat) to right (long repeat). The lanes for DM1-3 and DM1-4 have been rearranged for the LBR, SUN2, TMEM70, and TMEM38a blots to allow a presentation according to repeat length (indicated by black boxes). For original blots see supplemental material. **(B)** Immunofluorescence staining of primary control and DM1 myoblasts for emerin, LBR, Sun2 TMEM38a TMEM70 and Sun1. Scale bar 10 μm.

Due to the fact that some nesprin isoforms are up-regulated during muscle differentiation (Duong et al., 2014), we decided to test the expression of the nesprins in both myoblasts and myotubes of patients and controls (Figure 2). There are two skeletal muscle specific isoforms of nesprin 1 and 2, nesprin-1-alpha-2 (N1-α2, ~111 kDa) and nesprin-2-alpha-1 (N2-α1, ~72 kDa). Other detected bands are likely degradation products (Duong et al., 2014). For nesprin 1, we used two different antibodies: N1-α2 polyclonal and 8C3 monoclonal. In myoblasts both antibodies detect the possible giant isoform (300+ kDa) and the N1-α2 antibody detects in addition the muscle-specific 111 kDa band. The 8C3 antibody shows that nesprin1 giant is down-regulated in the DM1 patients and the N1-α2 antibody shows a similar result for the possible giant isoform, although not as clearly. In myotubes several additional bands are detected, but all are comparable to controls. However, there is a tendency for decreased expression of these isoforms with increased repeat length. Immunofluorescence staining with the N1-α2 antibody shows no obvious differences between controls and patients (Figure 2A).

For nesprin 2 we did not detect any expression changes between controls and patients in the myoblasts, but in myotubes there is also the tendency of a ~250 kDa band to decrease with increasing repeat length in the patients; however, as observed with the other proteins, this decrease is still within the range observed in controls. Immunofluorescence staining shows a weaker NE staining compared to nesprin 1 but no differences between controls and patients (Figure 2B).

## Discussion

Based on our prior observation of NE alterations in DM1 myoblasts (Meinke et al., 2018), we investigated NETs regarding their expression and localization to explore their possible contribution to these structural changes. In myoblasts we found no localization changes for any of the investigated NETs (emerin, LBR, SUN1, SUN2, TMEM38a, TMEM70, nesprin 1, and nesprin 2). However, this does not exclude a contribution of the NE in DM1. A study of NETs in Emery-Dreifuss muscular dystrophy (EDMD) primary myoblasts showed that NETs are inconsistent markers for this disease (Le Thanh et al., 2017) despite the fact that all identified EDMD causing mutations are in NE proteins. The missing NE staining of TMEM38a in myoblasts as well as the observed speckles inside the nucleus are consistent with other published data, although the NE localization could be confirmed in muscle fibers (Le Thanh et al., 2017). The same is probably the case for TMEM70. The observed tendency of reduced expression with increasing repeat length within the patients for SUN2, TMEM70 and to some degree as well emerin and the ~30 kDa band detected by the TMEM38a antibody might reflect the fact that not all DM1 myoblast nuclei have invaginations. Therefore, this might reflect changes in a subpopulation of cultured cells.

The decreased expression of nesprin 1 giant in DM1 myoblasts might be a contributory factor for the NE invaginations as nesprins are thought to form a network around the nucleus, thus contributing to its stability (Lu et al., 2012). However, it will be necessary to undertake further investigations to confirm this. The expression of smaller nesprin 1 and 2 isoforms during differentiation shows a similar tendency as the expression of the NETs SUN2, TMEM70, emerin and the possible TMEM38a isoform: reduced expression with increasing repeat length while still being in the range of the controls.

Overall the accumulation of small expression changes in several NETs could have an effect on NE mechanical stability, resulting in the NE invaginations observed in the patient cells. The expression changes observed for the nesprin 1 giant isoform could be contributory not only to the NE deformations but also to the disease phenotype, consistent with reported NE aberrations from nesprin 1 mutations linked to EDMD (Zhang et al., 2007) and spinocerebellar ataxia (Gros-Louis et al., 2007).

## Conclusion

Changes in NET expression levels may contribute to NE alterations observed in DM1 primary myoblasts. Several muscle NETs showed expression changes which appear to correlate with *DMPK*-repeat length. The giant isoform of nesprin 1 seems to be a good candidate for further analysis regarding its potential contribution to DM1 NE alterations and pathology.

## Author contributions

PM and BS contributed to the conception and design of the experiments. PM and SH wrote the manuscript, SH, LK, and SL performed the experiments.

### Conflict of interest statement

The authors declare that the research was conducted in the absence of any commercial or financial relationships that could be construed as a potential conflict of interest.

## References

[B1] Bione S. Maestrini E. Rivella S. Mancini M. Regis S. Romeo G.. (1994). Identification of a novel X-linked gene responsible for Emery-Dreifuss muscular dystrophy. Nat Genet. 8, 323–327. 10.1038/ng1294-323 7894480

[B2] Carrell S. T. Carrell E. M. Auerbach D. Pandey S. K. Bennett C. F. Dirksen R. T. (2016). Dmpk gene deletion or antisense knockdown does not compromise cardiac or skeletal muscle function in mice. Hum Mol Genet. 25, 4328–4338. 10.1093/hmg/ddw266 27522499 PMC5291200

[B3] Crisp M. Liu Q. Roux K. Rattner J. B. Shanahan C. Burke B.. (2006). Coupling of the nucleus and cytoplasm: role of the LINC complex. J. Cell Biol. 172, 41–53. 10.1083/jcb.200509124. 16380439 PMC2063530

[B4] De Antonio M. Dogan C. Hamroun D. Mati M. Zerrouki S. Eymard B.. (2016). Unravelling the myotonic dystrophy type 1 clinical spectrum: a systematic registry-based study with implications for disease classification. Revue Neurol. 172, 572–580. 10.1016/j.neurol.2016.08.003 27665240

[B5] Duong N. T. Morris G. E. Lam le T Zhang Q. Sewry C. A. Shanahan C. M.. (2014). Nesprins: tissue-specific expression of epsilon and other short isoforms. PLoS ONE 9:e94380. 10.1371/journal.pone.0094380 24718612 PMC3981789

[B6] Fu Y. H. Friedman D. L. Richards S. Pearlman J. A. Gibbs R. A. Pizzuti A.. (1993). Decreased expression of myotonin-protein kinase messenger RNA and protein in adult form of myotonic dystrophy. Science 260, 235–238. 8469976 10.1126/science.8469976

[B7] Fu Y. H. Pizzuti A. Fenwick R. G. Jr. King J. Rajnarayan S. Dunne P. W.. (1992). An unstable triplet repeat in a gene related to myotonic muscular dystrophy. Science 255, 1256–1258. 1546326 10.1126/science.1546326

[B8] Furling D. Coiffier L. Mouly V. Barbet J. P. St Guily J. L. Taneja K.. (2001). Defective satellite cells in congenital myotonic dystrophy. Hum. Mol. Genetics 10, 2079–2087. 10.1093/hmg/10.19.2079 11590125

[B9] Gros-Louis F. Dupre N. Dion P. Fox M. A. Laurent S. Verreault S.. (2007). Mutations in SYNE1 lead to a newly discovered form of autosomal recessive cerebellar ataxia. Nat Genet. 39, 80–85. 10.1038/ng1927 17159980

[B10] Harmon E. B. Harmon M. L. Larsen T. D. Paulson A. F. Perryman M. B. (2008). Myotonic dystrophy protein kinase is expressed in embryonic myocytes and is required for myotube formation. Dev Dynam. 237, 2353–2366. 10.1002/dvdy.21653 18729234

[B11] Harmon E. B. Harmon M. L. Larsen T. D. Yang J. Glasford J. W. Perryman M. B. (2011). Myotonic dystrophy protein kinase is critical for nuclear envelope integrity. J Biol Chem. 286, 40296–40306. 10.1074/jbc.M111.241455 21949239 PMC3220565

[B12] Holmer L. Worman H. J. (2001). Inner nuclear membrane proteins: functions and targeting. Cell Mol Life Sci. 58, 1741–1747. 10.1007/PL00000813 11766875 PMC11337314

[B13] Korfali N. Wilkie G. S. Swanson S. K. Srsen V. de Las Heras J. Batrakou D. G.. (2012). The nuclear envelope proteome differs notably between tissues. Nucleus 3, 552–564. 10.4161/nucl.22257 22990521 PMC3515538

[B14] Le Thanh P. Meinke P. Korfali N. Srsen V. Robson M. I. Wehnert M.. (2017). Immunohistochemistry on a panel of Emery-Dreifuss muscular dystrophy samples reveals nuclear envelope proteins as inconsistent markers for pathology. Neuromusc Disord. 27, 338–351. 10.1016/j.nmd.2016.12.003 28214269 PMC5380655

[B15] Lu W. Schneider M. Neumann S. Jaeger V. M. Taranum S. Munck M.. (2012). Nesprin interchain associations control nuclear size. Cell Mol Life Sci. 69, 3493–3509. 10.1007/s00018-012-1034-1 22653047 PMC11114684

[B16] Mastroyiannopoulos N. P. Chrysanthou E. Kyriakides T. C. Uney J. B. Mahadevan M. S. Phylactou L. A. (2008). The effect of myotonic dystrophy transcript levels and location on muscle differentiation. Biochem Biophys Res Commun. 377, 526–531. 10.1016/j.bbrc.2008.10.031. 18930030

[B17] Meinke P. Hintze S. Limmer S. Schoser B. (2018). Myotonic dystrophy - a progeroid disease? Front Neurol. 9:601. 10.3389/fneur.2018.00601 30140252 PMC6095001

[B18] Meinke P. Mattioli E. Haque F. Antoku S. Columbaro M. Straatman K. R.. (2014). Muscular dystrophy-associated SUN1 and SUN2 variants disrupt nuclear-cytoskeletal connections and myonuclear organization. PLoS Genet. 10:e1004605. 10.1371/journal.pgen.1004605. 25210889 PMC4161305

[B19] Meinke P. Schirmer E. C. (2016). The increasing relevance of nuclear envelope myopathies. Curr Opin Neurol. 29, 651–661. 10.1097/WCO.0000000000000359 27389815

[B20] Reddy S. Smith D. B. Rich M. M. Leferovich J. M. Reilly P. Davis B. M.. (1996). Mice lacking the myotonic dystrophy protein kinase develop a late onset progressive myopathy. Nat Genet. 13, 325–335. 10.1038/ng0796-325 8673132

[B21] Robson M. I. de Las Heras J. I. Czapiewski R. Le Thanh P. Booth D. G. Kelly D. A.. (2016). Tissue-specific gene repositioning by muscle nuclear membrane proteins enhances repression of critical developmental genes during myogenesis. Mol Cell 62, 834–847. 10.1016/j.molcel.2016.04.035. 27264872 PMC4914829

[B22] Rodriguez R. Hernandez-Hernandez O. Magana J. J. Gonzalez-Ramirez R. Garcia-Lopez E. S. Cisneros B. (2015). Altered nuclear structure in myotonic dystrophy type 1-derived fibroblasts. Mol Biol Rep. 42, 479–488. 10.1007/s11033-014-3791-4 25307018

[B23] Salpingidou G. Smertenko A. Hausmanowa-Petrucewicz I. Hussey P. J. Hutchison C. J. (2007). A novel role for the nuclear membrane protein emerin in association of the centrosome to the outer nuclear membrane. J. Cell Biol. 178, 897–904. 10.1083/jcb.200702026 17785515 PMC2064615

[B24] Udd B. Krahe R. (2012). The myotonic dystrophies: molecular, clinical, and therapeutic challenges. Lancet Neurol. 11, 891–905. 10.1016/S1474-4422(12)70204-1 22995693

[B25] Wenninger S. Montagnese F. Schoser B. (2018). Core clinical phenotypes in myotonic dystrophies. Front Neurol. 9:303. 10.3389/fneur.2018.00303 29770119 PMC5941986

[B26] Wilkie G. S. Korfali N. Swanson S. K. Malik P. Srsen V. Batrakou D. G.. (2011). Several novel nuclear envelope transmembrane proteinsidentified in skeletal muscle have cytoskeletal associations. Mol Cell Prot. 10:M110003129. 10.1074/mcp.M110.003129 20876400 PMC3016689

[B27] Zhang Q. Bethmann C. Worth N. F. Davies J. D. Wasner C. Feuer A.. (2007). Nesprin-1 and−2 are involved in the pathogenesis of Emery Dreifuss muscular dystrophy and are critical for nuclear envelope integrity. Hum Mol Genet. 16, 2816–2833. 10.1093/hmg/ddm238. 17761684

