# Supplementary figures and images for: Nuclear Envelope Transmembrane Proteins in Myotonic Dystrophy Type 1
Source: Front Physiol. 2018 Oct 30;9:1532. doi: 10.3389/fphys.2018.01532 (PMC6218431; doi:10.3389/fphys.2018.01532)

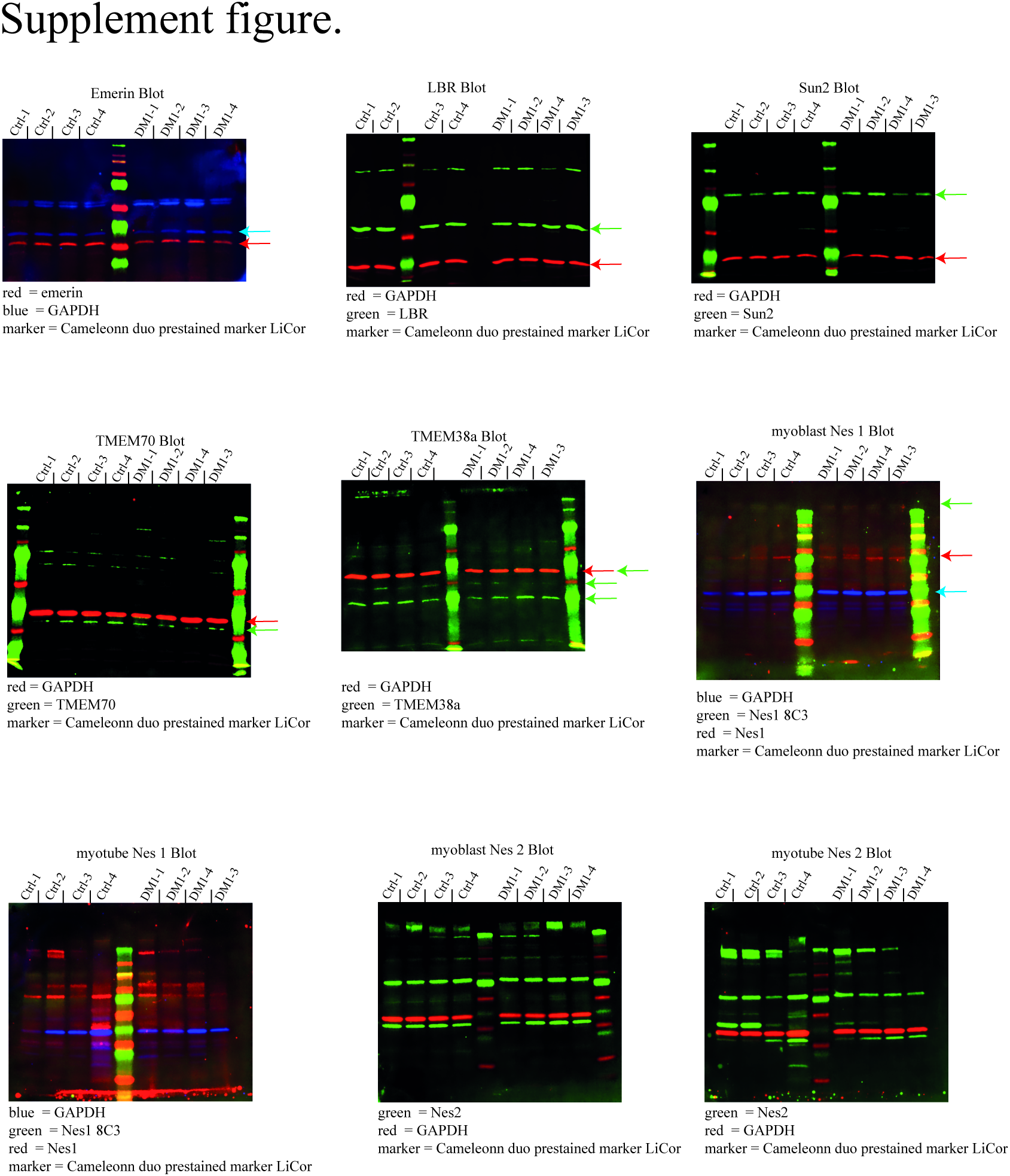

Supplement: Supplementary file 1 [file Image_1.TIF]
